# Selection of Cashmere Fineness Functional Genes by Translatomics

**DOI:** 10.3389/fgene.2021.775499

**Published:** 2022-01-04

**Authors:** Yu Zhang, Dongyun Zhang, Yanan Xu, Yuting Qin, Ming Gu, Weidong Cai, Zhixian Bai, Xinjiang Zhang, Rui Chen, Yingang Sun, Yanzhi Wu, Zeying Wang

**Affiliations:** ^1^ College of Animal Science andVeterinary Medicine, Shenyang Agricultural University, Shenyang, China; ^2^ International Business School and International Economics and Trade, Shenyang Normal University, Shenyang, China

**Keywords:** cashmere fineness, translatomics, Ribo-seq, COL6A5, liaoning cashmere goat

## Abstract

Cashmere fineness is an important index to evaluate cashmere quality. Liaoning Cashmere Goat (LCG) has a large cashmere production and long cashmere fiber, but its fineness is not ideal. Therefore, it is important to find genes involved in cashmere fineness that can be used in future endeavors aiming to improve this phenotype. With the continuous advancement of research, the regulation of cashmere fineness has made new developments through high-throughput sequencing and genome-wide association analysis. It has been found that translatomics can identify genes associated with phenotypic traits. Through translatomic analysis, the skin tissue of LCG sample groups differing in cashmere fineness was sequenced by Ribo-seq. With these data, we identified 529 differentially expressed genes between the sample groups among the 27197 expressed genes. From these, 343 genes were upregulated in the fine LCG group in relation to the coarse LCG group, and 186 were downregulated in the same relationship. Through GO enrichment analysis and KEGG enrichment analysis of differential genes, the biological functions and pathways of differential genes can be found. In the GO enrichment analysis, 491 genes were significantly enriched, and the functional region was mainly in the extracellular region. In the KEGG enrichment analysis, the enrichment of the human papillomavirus infection pathway was seen the most. We found that the *COL6A5* gene may affect cashmere fineness.

## Introduction

Cashmere goat is a unique animal husbandry resource in China (Cai et al., 2020; Jin et al., 2020), which has made outstanding contributions to the needs of the livestock textile industry and human society (Yang et al., 2020). The characteristics of cashmere show significant differences due to different goat species and regions (Jin et al., 2017). Cashmere goat has a double coat, with the primary hair follicles producing coarse hair and the secondary hair follicles producing fine hair (Dai et al., 2019). The cashmere thickness produced by the secondary hair follicles varies among individuals. Cashmere fiber is famous for being slender and soft. To improve the economic value of cashmere, the fineness of its fibers needs to be reduced, making cultivating high-yield and high-quality cashmere goat varieties the core element to improve the economic value of cashmere fiber. Cashmere fineness is a quantitative characteristic, which is determined by micro effective genes. Molecular breeding research in domestic and foreign studies have proved that the single nucleotide polymorphisms (SNPs) of *KAP* (Jin et al., 2011)*, RPL* (Mahata et al., 2012)*, FGF* (Liu et al., 2009)*, KRT* (Hui, 2020)*, PROP* (Zeng et al., 2011)*, MAF70* (Arranz et al., 2001), and *KIFI* (Li, 2009) genes are correlated with cashmere fineness. Many studies have identified some genes related to goat wool fiber growth characteristics, such as *DSG1, IGF-IR, KRTAPs, ILK,* and *KRTAP* genes (Rufaut et al., 1999; Jia et al., 2006; Yu et al., 2011; Yang et al., 2012; Liu et al., 2015). Full transcriptomics also proved that *NFKBIA, TCHH, COL1A1, CXCL8,* and *LTBP2* genes are closely related to cashmere fineness, and researchers have been looking for ways to improve the quantity and quality of cashmere (Bai et al., 2016; Zhang et al., 2018), as well as analyzing the key genes, signal pathways, and expression regulation level under different cashmere state diameters (Fu et al., 2020). Despite the efforts, all the genes regulating cashmere fineness from the translation level remain unknown. Due to the gap between the transcriptome and proteome in omics, only 20–40% of proteins in mammalian cells are determined at the transcription level (Tian et al., 2004; Cox et al., 2005). Current translatomics can make up the gap between the two. In the study of cashmere fineness, translatomics can be used to identify genes that are not translated into proteins at the transcriptional level. Translatomics provides new insights into gene expression (Courtes et al., 2013).

Translatomics is the sequencing and analysis of translated RNA molecules, which can accurately quantify the genes being translated, and compare the translation amount of different samples of genes under different physiological and pathological conditions or different treatments. At the same time, translation control is the key determinant of protein abundance, which in turn determines cell state (Sendoel et al., 2017). The gene expression process reveals that there are specific phenotypic characteristics among species, but their evolutionary process is uncertain outside the transcriptome. There are studies on coevolution at the level of mammalian transcriptome and translatome. Ribosome analysis and RNA sequencing are used to analyze the three organs of five mammals (human, macaque, mouse, opossum, and platypus) and birds (chicken), coevolutionary analysis (brain, liver, and testis) shows that translation regulation widely exists in different organs, especially in spermatogenic cell types of testis, and some genes evolve faster at the translatome level (Wang et al., 2020). Ribosomal analysis can also evaluate translation efficiency on a genome-wide scale, which has been previously proved in yeast (McManus et al., 2014), nematodes (Stadler and Fire, 2013), primate (Wang et al., 2018) cells, and hybrid mouse cells, it was also found that translation efficiency was a momentous predictor of protein level in mouse fibroblasts (Hou et al., 2015). These studies provide preliminary insights into the coevolution model of the transcriptome and translatome. Therefore, protein abundance seems to be mainly regulated by ribosomes, highlighting the importance of translation control (Gebauer and Hentze, 2004; Sonenberg and Hinnebusch, 2009). Using microRNA (miR-430) in zebrafish to investigate its translational repression and mRNA decay, we found that translation repression occurs before mRNA decay, which is induced by reducing the translation initiation rate, and that mRNA decay is induced by deadenylation. Besides, microRNA has been proposed to affect protein translation by reducing the rate of translation initiation (Bazzini et al., 2012). On the other hand, because the translatome studies RNA molecules being translated, which includes the RNA molecules that are traditionally considered noncoding, it can provide direct translation evidence for the study of these new translatable molecules (Gerashchenko et al., 2012). In addition, the lack of a strict direct correlation between gene and protein levels limits translation studies by combining the transcriptome and proteome. Considering the high cost associated with protein synthesis, the dominant role of translation regulation is meaningful. Therefore, it promotes the progress of translatomics technology (King et al., 1996; Kirkpatrick et al., 2005).

According to Tian Wenliang, the standard of cashmere fineness in China is: the diameter of coarse hairs: 16.0–18.5 μm and the diameter of fine hairs: 15.5–16.0 μm. In 2010, the cashmere of LCG was the thickest among all cashmere goats, and its diameter was 20.32 μm (Tian, 2015). It can be found that the main disadvantage of cashmere in LCG is that the cashmere is thicker. In this study, we found the key genes regulating cashmere fineness by using translatomics, and understood the regulatory relationship of related genes. We used Ribo-seq to test the fine skin samples and coarse skin samples of LCG, and found the key genes, differential genes, and co-expression genes related to cashmere fineness through GO function enrichment analysis and KEGG pathway enrichment analysis. These findings pave the way for the study of the regulation mechanism of cashmere fineness and the protection and cultivation of cashmere varieties.

## Materials and Methods

### Ethics Statement

The whole process of experiments was based on guidelines from the Animal Experimental Committee of Shenyang Agricultural University (Shenyang, China, 201906099).

### Sample Preparation

The sample collection was a crucial step for Ribo-seq since it was the starting point of library construction. Two groups of three Liaoning Cashmere Goats (LCGs) differing in cashmere fineness were used here, including three fine LCGs (14.32, 14.69, and 14.77 μm) and three coarse LCGs (17.23, 17.63, and 17.91 μm). These 2-year-old adult female Liaoning Cashmere Goats were reared under the same (sheep house, environment, management, nutrition) conditions. Skin samples were collected from the front edge of the left scapula, with a sample size of 2 cm^2^. Anesthetics were used to relieve the pain of goats.

### Library Construction for Ribo-Seq

To digest RNA other than RPFs, cell or tissue lysate was treated with unspecific endoribonuclease RNase ǀ. Isolation of monosomes was performed by size-exclusion chromatography with MicroSpin S-400HR columns. The RNA samples were then treated with an rRNA depletion kit to deplete the samples of as much rRNA contamination as possible before PAGE purification of the relatively short (20–38 nt) RPFs. Following PAGE purification, both ends of the RPF were phosphorylated and ligated with 5′ and 3′ adapters, respectively. Then the fragments were reversely transcribed to the cDNAs and amplified by PCR (Aeschimann et al., 2015).

After library construction, the concentration of the library was measured by The Qubit® 2.0 Fluorometer and adjusted to 1 ng/uL. An Agilent 2100 Bioanalyzer was deployed to examine the insert size of the acquired library. At last, the accurate concentration of the cDNA library was again examined using qPCR. Once the insert size and concentration of the library became identical, the samples could then be subjected for sequencing.

### Sequencing

After library preparation and pooling of different samples, the samples were subjected to Illumina sequencing. Commonly, the Ribo-seq uses PE150 (paired-end 150 nt) sequencing for 15 G raw data.

### Quality Control for Raw Data

Firstly, the initial data (in the format of FASTQ) and the adapter were processed to delete the 3′ ends sequence and obtain the clean data of Q20. The following analysis was based on the clean data.

### Mapping

Ribo-seq used TopHat2 for genome mapping. TopHat2 is an enhanced version of TopHat, using short read aligner Bowtie to align the RNA-seq reads to mammalian-sized genomes and analyzing the mapping result to identify splice junctions. TopHat2 allows variable-length indels with respect to the reference genome, which give it the ability to accurately align the transcriptomes in the presence of insertions, deletions, and gene fusions (Kim et al., 2013).

### Quantification of Gene Expression Level

Quantification of mapped results to gene level was carried out using HTSeq. HTSeq is a Python package that calculates the number of mapped reads to each gene (Anders et al., 2015). RPKM values were generated to represent the gene expression level of each specific gene. RPKM is the abbreviation of “Reads Per kilobase of transcript, per Million mapped reads,” which normalizes both sequencing depth and gene length (Gross et al., 2013).

### Differential Expression Analysis

For samples with biological replicates, the DESeq2 R package (1.14.1) was used for differential expression analysis. DESeq2 provides statistical routines for determining differential expression in digital gene expression data using a model based on negative binomial distribution (Wang et al., 2010). The resulting P-values were adjusted using the Benjamini and Hochberg’s approach for controlling the false discovery rate. Genes with *p* < 0.05 found by DESeq2 were assigned as differentally expressed (Tang et al., 2007).

### GO and KEGG Enrichment Analysis

Gene Ontology (GO) is a major bioinformatics initiative to unify the representation of gene and gene product attributes across all species. GO covers three domains: cellular component, molecular function, and biological process. KEGG (Kyoto Encyclopedia of Genes and Genomes) is a collection of databases dealing with genomes, biological pathways, diseases, drugs, and chemical substances (Kanehisa et al., 2008). In the KEGG pathway database, the wiring diagram database, is the core of the KEGG resource. It is a collection of pathway maps integrating many entities including genes, proteins, RNAs, chemical compounds, glycans, and chemical reactions, as well as disease genes and drug targets, which are stored as individual entries in the other databases of KEGG.

### P- Site Analysis

Identifying the A- and P-site locations on ribosome-protected mRNA fragments from Ribo-Seq experiments was a fundamental step in the quantitative analysis of transcriptome-wide translation properties at the codon level. The P-site (for peptidyl) is the second binding site for tRNA in the ribosome. During protein translation, the P-site holds the tRNA which is linked to the growing polypeptide chain. When a stop codon is reached, the peptidyl-tRNA bond of the tRNA located in the P-site is cleaved releasing the newly synthesized protein. Since translation occurs at the A- and P-sites, the identification of these sites was critical to address translation-related questions (Ahmed et al., 2019). Novogene used the Ribocode package to analyze the P-site using Ribo-seq data (Xiao et al., 2018).

### uORF Analysis

An upstream Open Reading Frame (uORF) is an Open Reading Frame (ORF) within the 5′ untranslated region (5′UTR) of an mRNA. uORFs can regulate eukaryotic gene expression. Associated with mRNA-seq, all identified ORFs by Ribo-seq were classified. Ribotape was then used to analyze the motif of translated/untranslated uORFs, which can be used to study the base composition bias of uORF sequences.

## Result

### Quality Control of Sequencing Data of Six LCGs

Through high-throughput sequencing, the raw reads sequences of fine LCG samples accounted to 50666392, 50286756, and 52749309. Low quality data accounted for about 0.21%. The rest were clean reads. The percentage of sequencing sequences that could be located on the genome was about 88.86%. The average percentage of sequencing sequences with unique alignment positions on the reference sequence was about 46.28%. About 99.06% of the base group had a mass value greater than 20. The average proportion of filtered rRNA to total clean reads was about 76.51%. The average proportion of filtered tRNA in the total number of clean reads was about 4.33%.

The raw reads sequences of coarse LCG samples accounted to 49894812, 51599006, and 59901380. Low quality data accounted for about 0.21%. The rest were clean reads. The percentage of sequencing sequences that could be located on the genome was about 81.64%. The average percentage of sequencing sequences with unique alignment positions on the reference sequence was about 39.10%. About 99.03% of the base group had a mass value greater than 20. The average proportion of filtered rRNA to total clean reads was about 76.66%. The average proportion of filtered tRNA in the total number of clean reads was about 3.77% (Table 1).

**TABLE 1:** Quality control table of fine type and coarse type data of LCG.

Sample	Raw reads	Low quality	Clean reads	Total mapped	Uniquely mapped	Q20 (%)	rRNA reads	tRNA reads
FT LCG 1	50666392	99284 (0.20%)	50431480	7931912 (88.92%)	4235822 (47.49%)	99.10	38526496 (76.39%)	2434096 (4.83%)
FT LCG 2	50286756	87865 (0.17%)	50051017	8231481 (88.65%)	3621884 (39.01%)	99.12	37090706 (74.11%)	3282429 (6.56%)
FT LCG 3	52749309	129738 (0.25%)	51299342	6667995 (89%)	3922342 (52.35%)	98.97	40540273 (79.03%)	818312 (1.60%)
CT LCG 4	49894812	146884 (0.29%)	47332796	3722651 (71.99%)	1381041 (26.71%)	98.81	37754715 (79.76%)	210679 (0.45%)
CT LCG 5	51599006	90692 (0.18%)	51323088	7626758 (87.43%)	4235618 (48.55%)	99.13	37502937 (73.07%)	3121300 (6.08%)
CT LCG 6	59901380	100832 (0.17%)	59536550	7563025 (85.51%)	3718500 (42.04%)	99.15	45932536 (77.15%)	2854453 (4.79%)

FT LCG 1 is the fine Liaoning Cashmere Goat No. 1 sample, FT LCG 2 is the fine Liaoning Cashmere Goat No. 2 sample, FT LCG 3 is the fine Liaoning Cashmere Goat No. 3 sample, CT LCG 4 is the coarse Liaoning Cashmere Goat No. 4 sample, CT LCG 5 is the coarse Liaoning Cashmere Goat No. 5 sample, and CT LCG 6 is the coarse Liaoning Cashmere Goat No. 6 sample; Q20 is the percentage of bases with phred values greater than 20 in the total bases.

**FIGURE 1:**
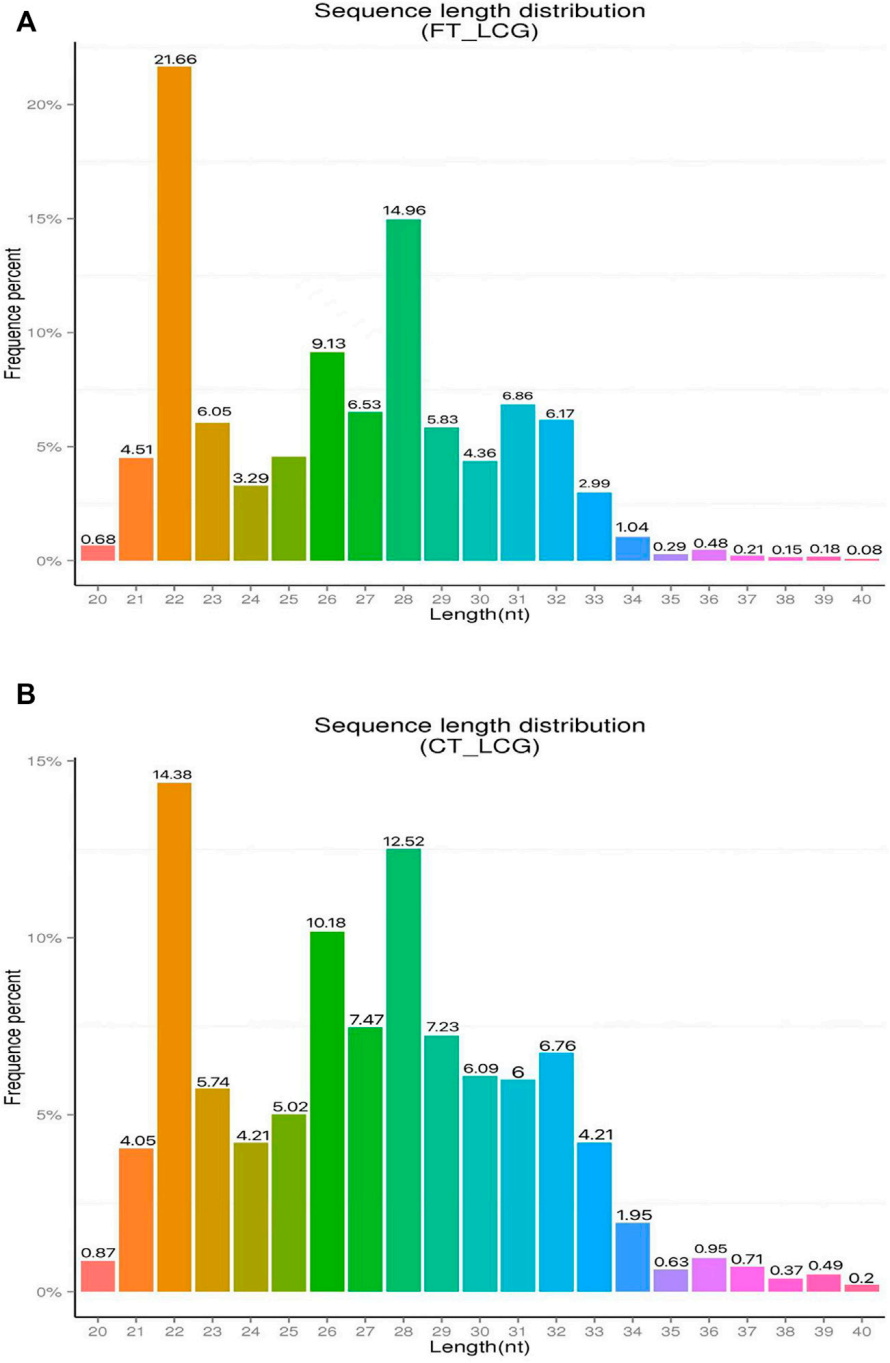
Statistical chart of clean reads length enrichment of Ribo-seq in LCG. The abscissa is the enrichment length and the ordinate is the percentage of enrichment frequency. **(A)** Sample of fine LCG. **(B)** Sample of coarse LCG.

**FIGURE 2:**
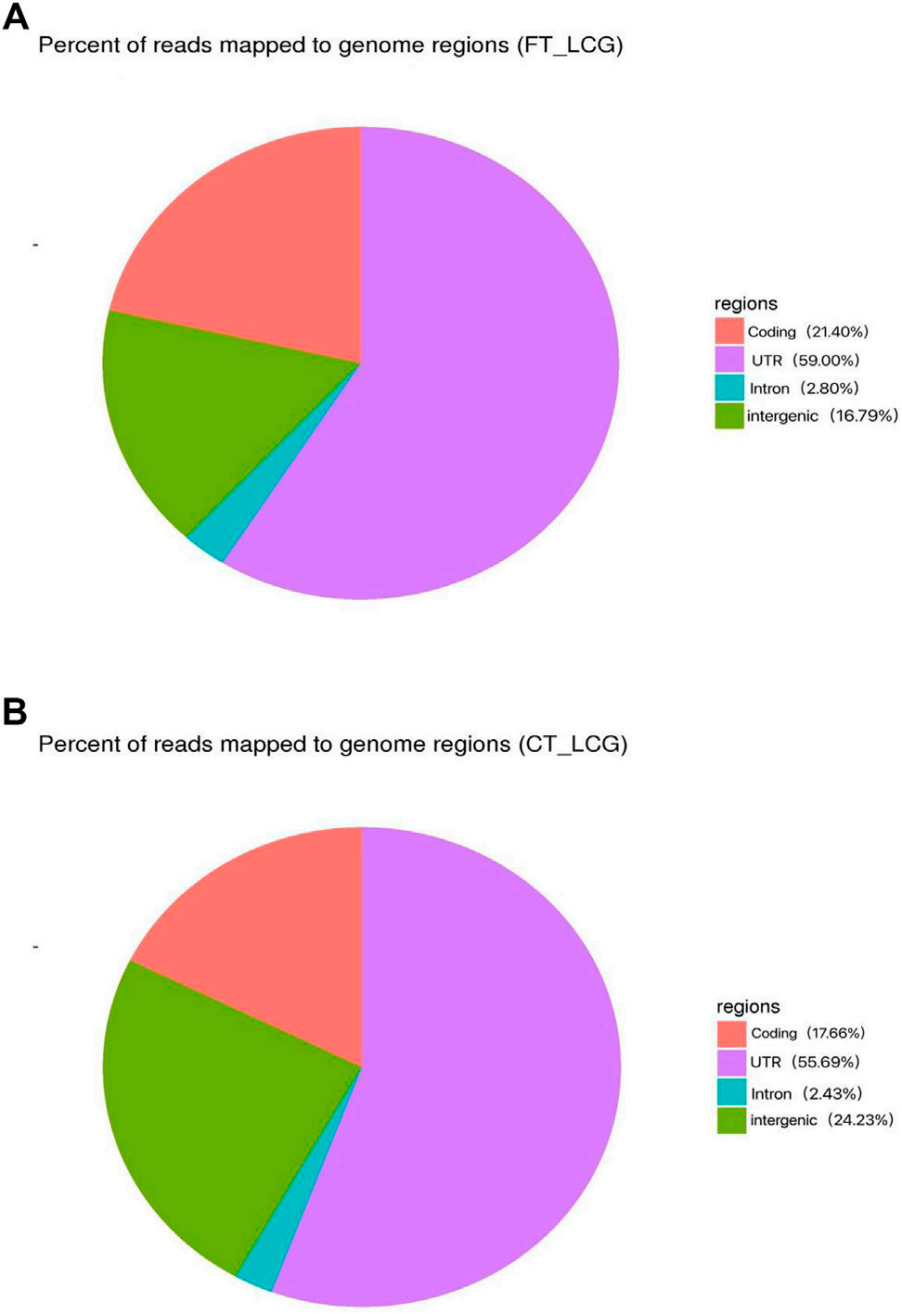
Distribution of reads in different regions of the reference genome. Red indicates the coding area. Purple indicates the UTR area. Green indicates the intergenic area. Blue indicates the intron area. **(A)** Sample of fine LCG. **(B)** Sample of coarse LCG.

The effect of experimental enrichment can be evaluated by counting the length of ribosome protected RNA fragments (RPFs). The length statistics of Ribo-seq clean reads of fine and coarse samples of LCG showed that when the enrichment length was 22 nt, the enrichment frequency was the highest, 21.66 and 14.38%, respectively (Figures 1A,B). The genomic region sequencing distribution is shown in the figure: the coding region of fine Liaoning cashmere goat accounted for 21.40%, the UTR region accounted for 59.00%, the intron region accounted for 2.80%, and the intergenic region accounted for 16.79% (Figure 2A). The coding region of coarse Liaoning cashmere goat accounted for 17.66%, the UTR region accounted for 55.69%, the intron region accounted for 2.43%, and the intergenic region accounted for 24.23% (Figure 2B).

### Quantitative Analysis and Distribution of Gene Expression in Six LCGs

The translation level of a gene protein is directly reflected by the abundance of ribosome binding on its corresponding transcript. The higher the binding abundance is, the higher the level of gene translation is. We quantitatively analyzed the gene expression level of the top 30 genes in each sample, as shown in Table 2.

**TABLE 2:** Quantitative analysis table of the first 30 gene expression levels of each sample.

Gene name	FT LCG 1	FT LCG 2	FT LCG 3	CT LCG 4	CT LCG 5	CT LCG 6
*LOC108638395*	445319	336977	1344217	574326	742089	514676
*MIR148A*	243740	359838	356349	26236	222275	342894
*COL1A1*	22968	46391	2220	4944	23410	30471
*COL1A2*	14548	28783	1640	2639	14371	17909
*EEF1A1*	451	716	208	105	395	337
*MIR10B*	243316	172218	204214	10272	113416	170024
*FTH1*	383	727	49	99	442	291
*COL3A1*	7095	19128	1036	1499	7544	10158
*MIRLET7I*	97833	118750	141902	44311	158213	56831
*LOC108635080*	157034	98967	135504	102991	47625	62559
*LOC102184404*	5989	7402	1133	392	6438	12665
*KRT5*	6949	13975	3529	1867	4549	7209
*MIR99A*	124457	66498	46951	10679	69591	180316
*TCHH*	4054	6942	1962	504	1893	4115
*KRT14*	4814	8188	1586	843	2640	4205
*FASN*	32449	4204	423	1209	9573	6267
*LOC102185436*	1517	1742	510	383	906	1625
*LOC102177231*	6496	10901	2395	1128	4154	5305
*MIR26A*	92770	73542	122982	7897	34599	79360
*LOC102184223*	2180	2251	646	404	1482	2195
*KRT25*	3583	6185	1085	662	2790	3315
*RPL4*	167	175	30	17	142	136
*RPS8*	141	219	24	22	150	100
*MIR126*	75155	59465	76507	8566	59254	55597
*TPT1*	73	72	42	52	74	71
*SPARC*	5707	10185	875	1213	4087	5190
*KRT10*	1490	3452	173	46	359	628
*DSP*	2491	4369	739	282	1181	2158
*RPLP0*	299	323	113	67	271	266
*COL6A5*	50	682	805	39	28	37

**FIGURE 3:**
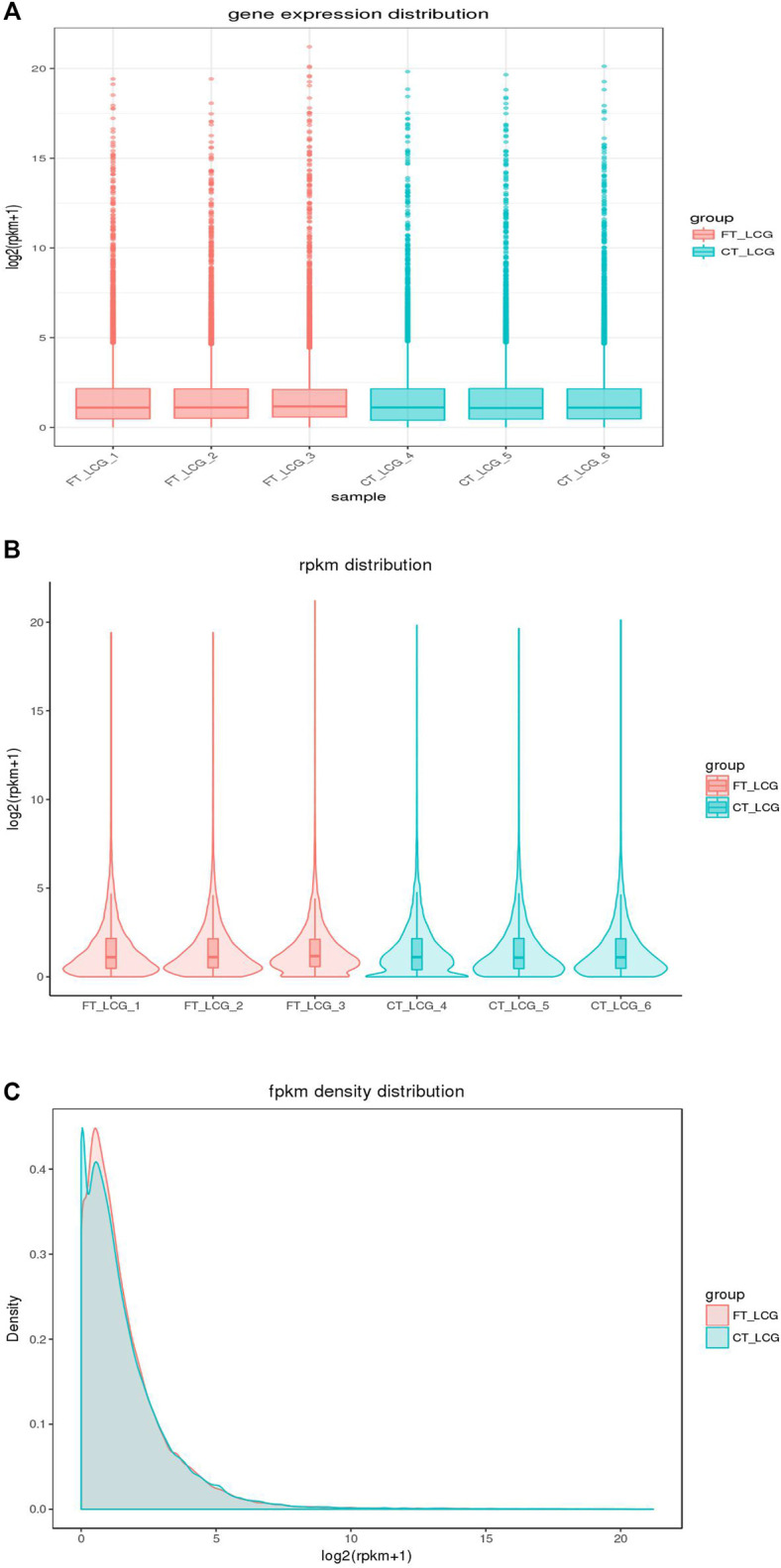
Distribution of gene expression in fine and coarse type LCGs. The abscissa in the graph is the sample name, and the ordinate is log2 (RPKM+1). The fine LCG sample is shown in pink, and the coarse LCG sample is shown in blue. The box graph of each region has five statistics (the maximum, upper quartile, median, lower quartile, and minimum from top to bottom).

In addition to the true translation level, the reads count was positively correlated with the sequencing depth. Generally, the gene expression value is not expressed by reading count, but by RPKM, which corrects the sequencing depth and gene length successively. After calculating all gene expression values (RPKM) of each sample, we showed the distribution of gene expression levels of different samples by box graph. From the box graph, we can see that there were differences in the expression levels of all the genes detected among the six samples, and the box graph of sample 3 was smaller than that of other samples, so we can see that the differences between the genes detected in sample 3 and other samples were more obvious. At the same time, obvious different genes were found between the two groups. The coincidence of the two groups was the co-expressed genes, and the noncoincidence was the differentially expressed genes (Figures 3A–C).

### The Statistics of Differential Genes Were Carried out, and the Volcanic Map and Cluster Map Were Drawn

After the quantitative analysis of gene expression, it is necessary to conduct statistical analysis on the expression data, and screen the genes with significantly different expression levels in different states. Some differentially expressed genes are shown in Table 3.

**TABLE 3:** Partial up-down regulation difference gene table between fine type and coarse type LCGs.

Gene name	FT LCG	CT LCG	P-value	Up/down
*COL6A5*	513.0210584	35.10807307	1.09E-06	Up
*ITIH4*	13.30490937	0.652476505	8.14E-06	Up
*LOC102173569*	19.27928687	2.77555756	1.16E-05	Up
*LOC102173761*	21.50257982	2.956095588	1.34E-05	Up
*CX3CR1*	11.42687495	0.597894581	1.38E-05	Up
*LOC102183314*	14.40120352	0.597421578	2.00E-05	Up
*LOC108638245*	13.94060036	0.324997368	2.54E-05	Up
*SCIN*	54.00576352	12.50050163	3.37E-05	Up
*MIR223*	526.9564919	77.06293267	6.86E-05	Up
*KRT2*	80.95366623	6.940780142	7.45E-05	Up
*LOC102181854*	11.9493693	0.668851595	0.000179452	Up
*SPI1*	7.221664344	2.77555756	0.000211171	Up
*TNC*	180.2254246	12.67495436	0.000214188	Up
*LTF*	49.98989748	6.732485479	0.000223432	Up
*SHANK3*	5.344749546	2.77555756	0.000234583	Up
*LOC102181202*	95.51666376	24.59851436	0.000284781	Up
*AGP*	8.736937811	2.77555756	0.000318954	Up
*LOC102185525*	92.67098586	15.49360806	0.00039787	Up
*LOC102191415*	34.15538801	3.040163575	0.000861835	Up
*BCL2A1*	8.331100162	0.977471879	0.001012572	Up
*ZNF550*	3.50939654215679	17.2539274369117	0.000425155705185925	Down
*MIOX*	0.178346971505201	6.58571066698514	0.000550580885740381	Down
*LOC108636746*	20.2230313525199	71.2478009465496	0.000575441389747231	Down
*LOC102175702*	2.77555756156289E-17	4.81857339537376	0.00092949091732039	Down
*LOC102168522*	0.942200471766223	7.50055476502101	0.00126514633141028	Down
*MIR9*	9.82710757202138	80.2714587053271	0.00139800098028482	Down
*MIR671*	5.33927118533789	56.4684485860653	0.00147473232541579	Down
*SDC3*	17.3545579173651	81.4319352084731	0.00162620276767528	Down
*LHX1*	1.34518325391645	8.62420593115666	0.00167178598923491	Down
*ABHD15*	5.12668482061614	49.4793752852943	0.00203138254923832	Down
*LOC102173583*	1.26347479869531	8.90431324840639	0.00210487578853974	Down
*LOC108634715*	0.573483698205509	7.36640964136039	0.00238509701852834	Down
*LOC108634352*	0.563608783979002	5.76016723909547	0.00260838978865612	Down
*LOC102190399*	0.178346971505201	4.31371001374939	0.00265357125604965	Down
*LOC102188887*	4.46437256485464	16.6419755873072	0.00330633387847916	Down
*CHD3*	40.3297349602123	203.461408220537	0.00342774246368716	Down
*LOC108638293*	9.2957498124219	30.6384923277523	0.00358064723751997	Down
*RARRES1*	43.7517203353152	121.773870596007	0.00366888299547616	Down
*CUX2*	0.383840891809286	6.11577320580318	0.00368401375810571	Down
*SLC25A11*	12.9565771393252	35.6648973518473	0.00369472188184243	Down

**FIGURE 4:**
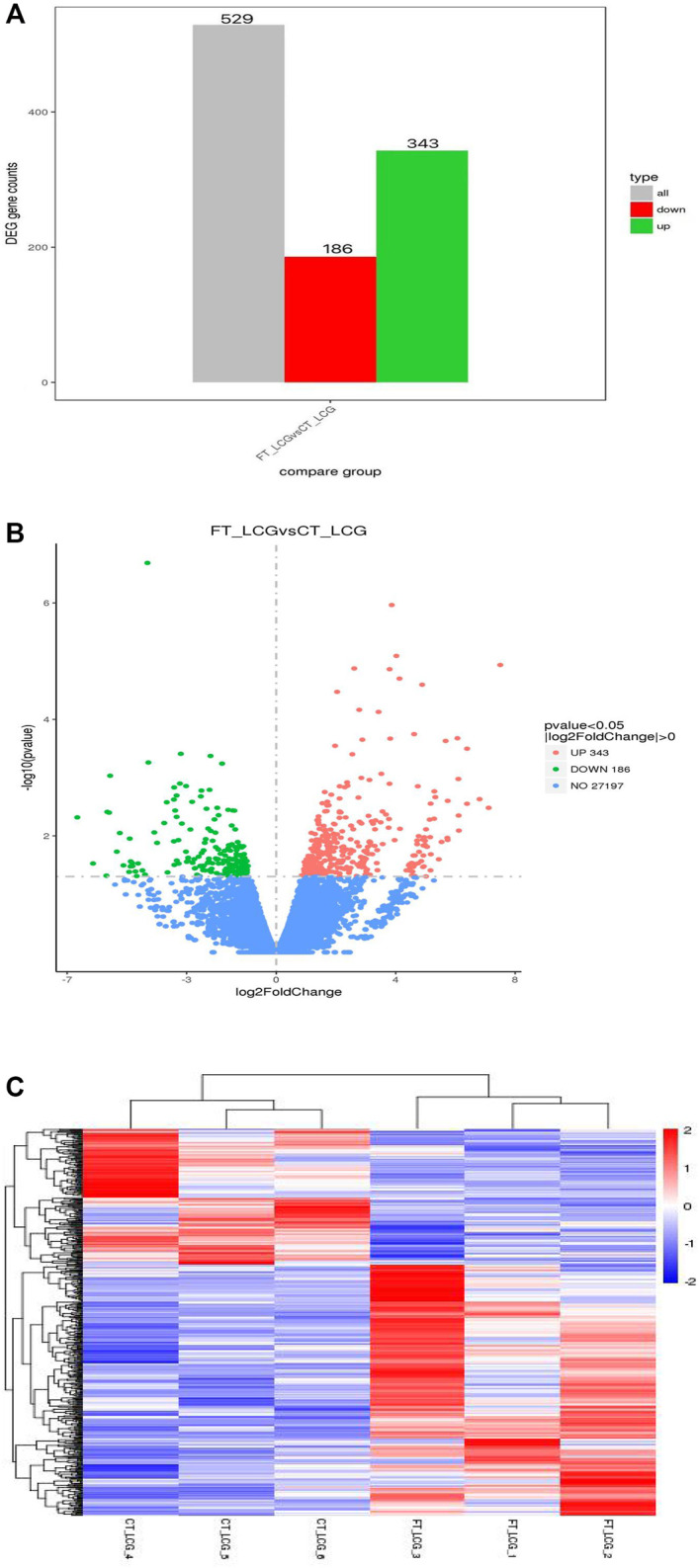
Statistical chart of the number of different genes between fine type and coarse type LCG. **(A)** The abscissa of the histogram is divided into two groups: fine type and coarse type, the ordinate is the gene counts. **(B)** In the volcanic map, the abscissa represents the log2foldchange of gene expression in the treatment and control groups, and the ordinate represents the significant level of gene expression difference between the treatment and control groups, in which the upregulated genes are indicated by red dots and the downregulated genes are indicated by green dots. **(C)** Gene expression cluster map of fine and coarse type LCG. In the graph, red indicates high gene expression and blue indicates low gene expression. The abscissa is the sample name, and the ordinate is the normalized value of RPKM.

The fine type and coarse type LCGs were compared by histogram and volcanic map, we can see that there were 27197 coexpressed genes and 529 differential genes, of which 343 were upregulated and 186 were downregulated. The clustering graph compared the gene expression of three samples of coarse LCG and three samples of fine LCG. Therefore, it can be seen that the overall gene expression trend of the three samples of coarse LCG was significantly different from that of the three samples of fine LCG. The genes with high expression in fine LCG had lower expression in coarse LCG, and the genes with low expression in fine LCG had higher expression in coarse LCG (Figures 4A–C).

### GO Enrichment Analysis and KEGG Enrichment Analysis of Differential Genes

**FIGURE 5:**
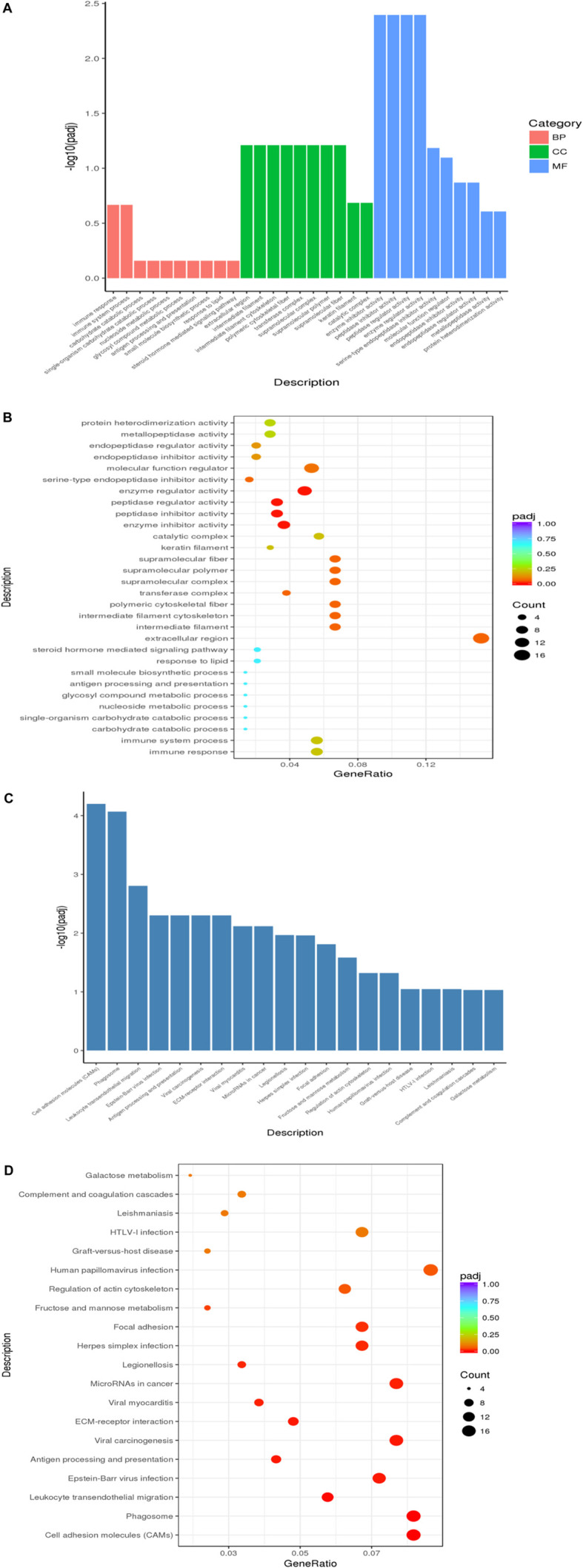
Enrichment analysis in LCG. **(A)** GO enrichment analysis histogram, the abscissa is the GO term, and the ordinate is the significance level of GO term enrichment. Height was positively correlated with the significance. Different colors represent BP, CC, and MF. **(B)** Scatter plot of enrichment analysis, the horizontal coordinate in the Panel is the ratio of the number of different genes annotated to the GO term and the total number of different genes. The ordinate is the GO term, the size of points represents the number of genes annotated to the GO term, and the level of enrichment varies from purple to red. **(C)** KEGG enrichment analysis histogram, the horizontal coordinate in the panel is the KEGG pathway, and the longitudinal coordinate is the significance level of channel enrichment, and the height of the histogram indicates the degree of enrichment. **(D)** Scatter plot of KEGG enrichment analysis, the abscissa is the ratio of the number of differential genes annotated to the KEGG pathway to the total number of differential genes. The ordinate is the KEGG pathway. The size of the dot represents the number of genes annotated to the KEGG pathway. And the level of enrichment varies from purple to red.

We Used ClusterProfiler Software (Yu et al., 2012) for analysis, through GO enrichment analysis, we selected the first 30 for analysis, and found 491 significantly enriched genes, including biological process (BP, 257), cell composition (CC, 64), and molecular function (MF, 170). The function was mainly in the extracellular region (Figures 5A,B). From the results of KEGG enrichment, the most significant 20 KEGG pathways were selected, and the most enriched pathway was the human papillomavirus infection pathway (Figures 5C,D).

### P-Site Analysis of Six LCGs

**FIGURE 6:**
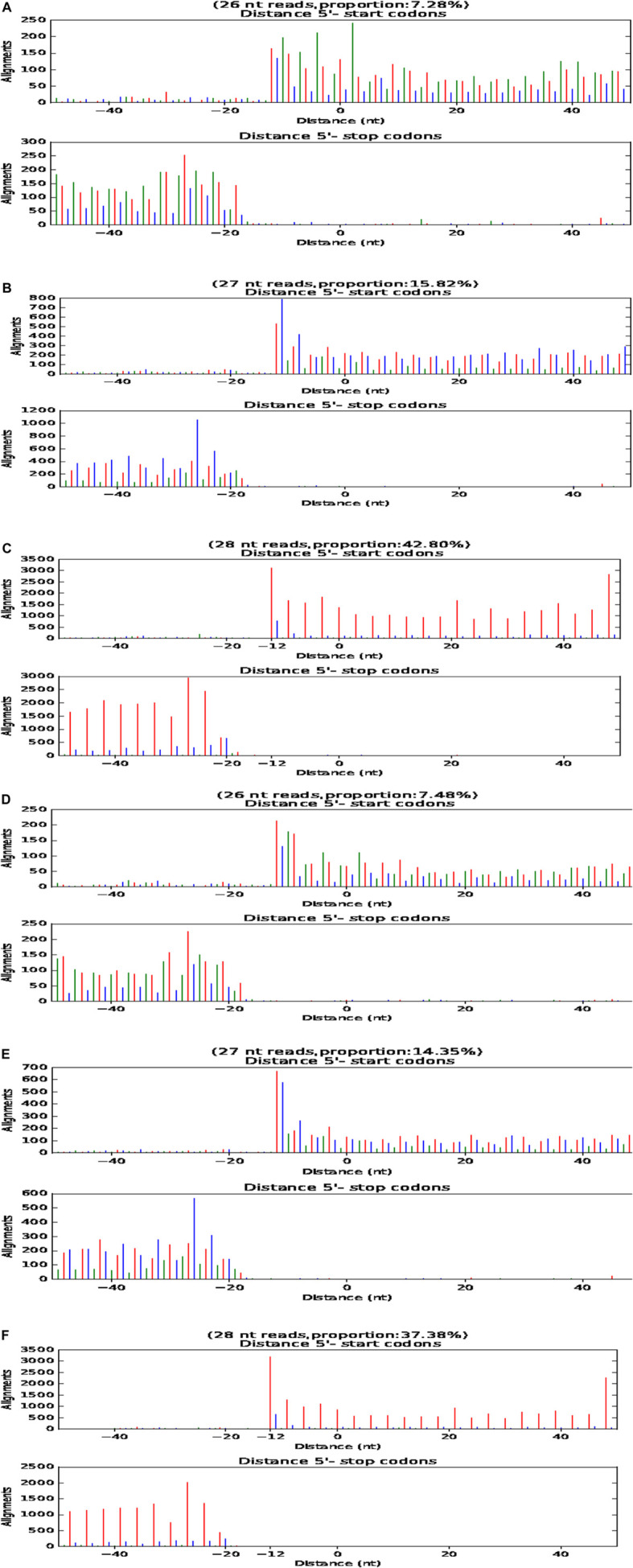
P-site map of fine type and coarse type LCG. The abscissa is the distance from the start code or stop code, and the ordinate is the reads on the comparison. **(A–C)** P-site map of three fine type samples of LCG. **(D–F)** P-site map of three coarse samples of LCG.

During translation, ribosomes move in the unit of codon length (3 nt) relative to RNA. Therefore, based on P-site, RPF fragments from normal translation should be periodically distributed on RNA. The translation starts at 12 nt upstream of the initiation codon, and the distance from the termination codon 15 nt disappears gradually. This is direct evidence of whether an RNA is translated. The length of RNA fragments protected by the ribosome from the initial codon to the termination codon of three samples of LCG was 26, 27, and 28 nt, respectively (Figure 6).

### ORF Analysis of Six Samples of LCGs

**FIGURE 7:**
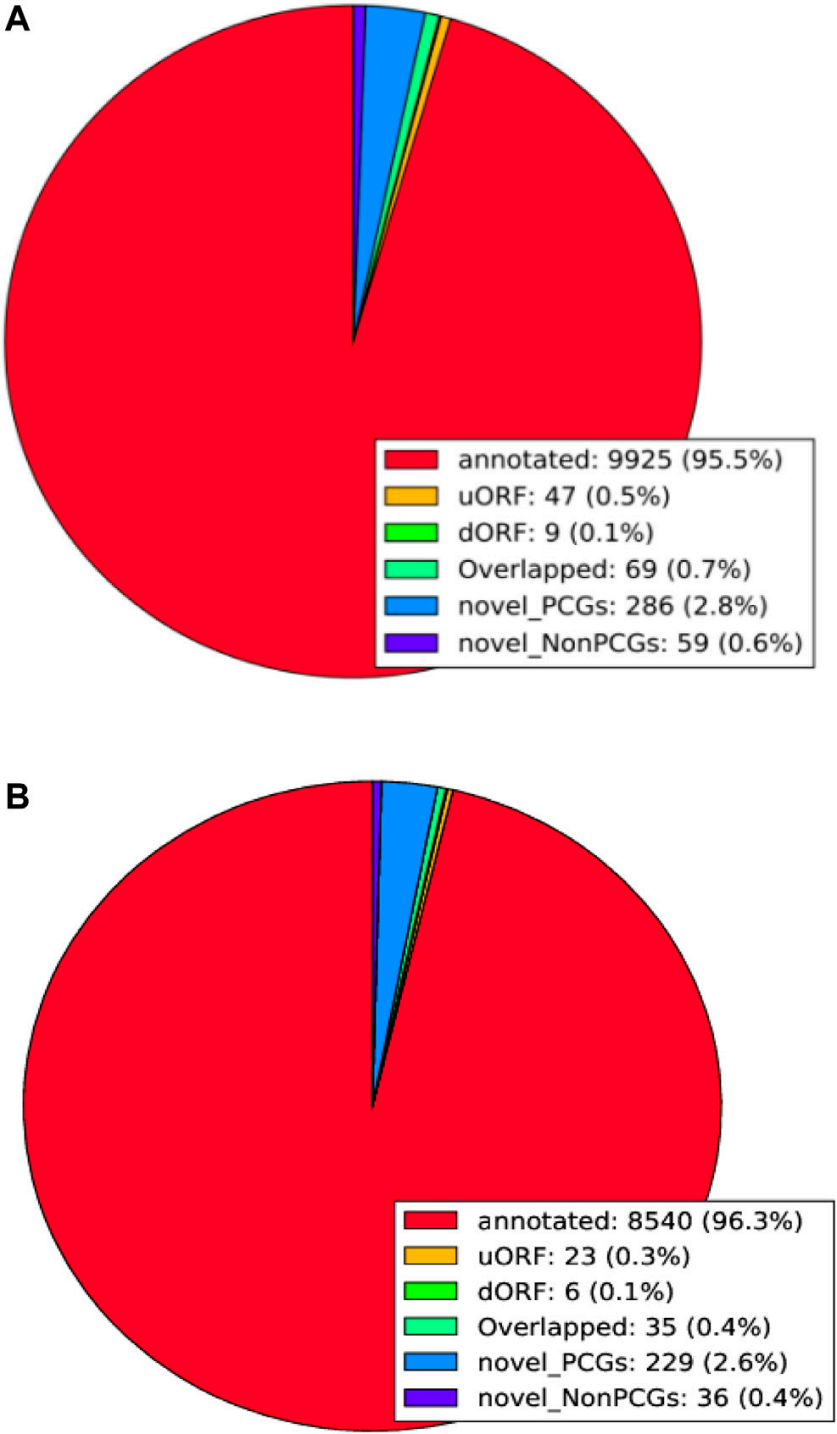
ORF analysis of LCG. **(A)** ORF analysis of fine LCG. **(B)** ORF analysis of coarse LCG.

In the samples of fine LCG, 9925 genes were transformed into protein during the translation process, accounting for 95.5% of the total. Among them, 47 uORF (0.5%) and 9 dORF (0.1%) could be translated into protein. There were 69 overlapping genes (0.7%), 286 novel-PCGs (2.8%), and 59 novel-NonPCGs (0.6%) (Figure 7A). There were 8540 genes (96.3%) transformed into protein in coarse LCG samples during translation, among which 23 genes (0.3%) were uORF and 6 genes (0.1%) were dORF. There were 35 overlapping genes (0.4%), 229 novel-PCGs (2.6%), and 36 novel-NonPCGs (0.4%) (Figure 7B).

## Discussion

LCG is unique in China, which produces a large number of high-quality cashmere fibers. Moreover, China is one of the largest cashmere producing countries in the world, which has made great contributions to the fiber industry and textile industry, and plays an indispensable role in the global cashmere industry (Zheng et al., 2020). But at present, the pursuit of cashmere fineness is increasing, and the cashmere fineness of LCG is still showing a relatively coarse trend, and quality cashmere products are still insufficient. Reducing cashmere fineness is an important issue (Zheng et al., 2019). Ribosome profiling is a mature method to identify translation regions by high-throughput sequencing, which fills the gap between RNA sequencing and proteomics, and has become a powerful tool for gene expression (Calviello and Ohler, 2017). Ribo-seq can not only measure the translation efficiency according to the relative abundance of ribosomes on transcripts, but also reveal the dynamic and local regulation of different translation stages according to the location information of footprints on transcripts (Li et al., 2020).

However, little is known about the issue of cashmere fineness of LCG by Ribo-seq sequencing in translatomics. In this study, we selected six adult female Liaoning Cashmere Goats (LCGs) with different cashmere fineness (divided into two groups). The coarse Liaoning Cashmere Goat sample group was the reference group, and the fine Liaoning Cashmere Goat sample group was the experimental group. The classification of groups was based on the phenotypic determination of cashmere fineness (cashmere fineness analyzer, sirolan technology, Australia). A total of 529 differentially expressed genes were identified by Ribo-seq sequencing, of which 343 were upregulated and 186 were downregulated. And the enrichment length of mRNA fragments was 22 nt.

*COL6A5* (formerly known as *COL29A1*) is a member of the collagen superfamily. The gene is located on the long arm of chromosome 3 (Strafella et al., 2019), with a length of 8742. It is a protein-coding gene and is considered to be an extracellular matrix protein, accounting for 30% of the total mammalian protein (Haq et al., 2019). *COL6A5* was found in the epithelial tissues of lung and gastrointestinal tract (Fitzgerald et al., 2008), but the highest expression was found below the dermal epidermal junction and around the reticular dermal vessels (Sabatelli et al., 2011). It was found that *COL6A5* fibroblasts existed in atopic dermatitis skin, but not in healthy tissues (He et al., 2020). The *COL6A5* gene is not only associated with skin inflammation, but also with cancer. It has been confirmed that the *COL6A5* gene is significantly associated with the overall survival rate of patients with esophageal squamous cell carcinoma (ESCC). The overall survival rate of ESCC patients with low expression of the *COL6A5* gene is poor, which may become a diagnostic marker of ESCC as a collagen gene (Li et al., 2019). Abundant type ⅵcollagen in lung tissue α5 (*COL6A5*), rs13062453, rs1497305, and rs77123808 of *COL6A5* polymorphism are associated with lung cancer risk in Chinese Han population, and the overall survival rate of patients with low expression of the *COL6A5* gene is poor (Duan et al., 2020). These studies can infer that the *COL6A5* gene may play a role in changing the hair follicle and cashmere fineness of LCG.

Because of that and because another gene from the same family, COL1A1, is known to have an impact on changing cashmere fineness (Wang et al., 2021), we hypothesize that COL6A5 is a candidate gene for future studies regarding cashmere fineness. Studies have shown that *COL6A5* is associated with familial chronic neurotrophic itching (Martinelli-Boneschi et al., 2017). It has been found that *COL6A5* expression in the papillary dermis and the surrounding dermis of the patients is reduced (Weisshaar and Dalgard, 2009; Ständer et al., 2010), and the incidence rate increases with age. This is the first time that the link between the *COL6A5* gene and chronic pruritus has been revealed. Some studies have found a link between *COL6A5* variants, reduced bone mass, dyspnea, and giant cell arteritis (Wang et al., 2016). In psoriasis (Ps) and psoriatic arthritis (PsA), bioinformatics analysis revealed that *COL6A5* and *COL8A1* participate in the altered proliferation and angiogenesis pathways in Ps/PsA, participate in inflammatory response together with *miR-146a,* and participate in the common and different biological pathways of Ps and PsA (Caputo et al., 2020).

The collagen gene may be closely related to the PI3K/Akt/mTOR pathway, p53 pathway, apoptosis, and cell cycle. *COL1A1* and *FGF10* genes are also enriched in the PI3K/Akt/mTOR pathway. *COL1A1* can regulate the growth of alpaca wool fiber, and *FGF10* can prolong the growth period of mouse hair follicles and promote hair growth (Maiese, 2015; Huang et al., 2017; Mendoza et al., 2019). Therefore, we take *COL6A5* gene as a candidate gene for cashmere fineness research in the future.

In living organisms, ribosomes synthesize proteins in the process of translation, and translation regulation itself goes beyond the three processes of transcription, mRNA degradation, and protein degradation. Like other omics, translatomics analyzes all components in the translation process, and also includes the study of mRNAs, ribosomes, tRNAs, regulatory RNAs, and newborn peptide chains (Zhao et al., 2019). Meanwhile, the correlation between the transcriptome and proteome is usually very poor in omics data, because the phenomenon of post transcriptional regulation, translation control, and other factors such as frameset translation is common. Translational sequencing can accurately quantify the genes being translated, and indirectly detect the protein expression from the genomic level, indicating the real situation of gene transcription and expression in biological samples. In addition, by comparing the gene translation differences between different samples, we can reveal the molecular mechanism of related physiological and pathological differences. Translatomics is a bridge between transcriptomics and proteomics. The multi omics analysis of translatomics can better study the mechanism of translation regulation. By analyzing the correlation between translatomics and transcriptomics, we can study the change of translation rate within genes, compare the relationship between gene transcription and translation, and study the difference of gene translation efficiency under different physiological and pathological conditions by calculating gene translation efficiency, to explain its molecular mechanism. The association analysis of translatomics and proteomics can study the relationship between transient translation and protein accumulation, assist proteome identification, provide evidence of gene translation, and indirectly determine the proteins expressed in biological samples.

It is critical to accurately investigate the underlying mechanisms of miRNA translation inhibition, and analyze the effect of post transcriptional regulation and RNA modification on gene translation. It is generally believed that the gene that can encode a protein with a length of more than 100 amino acids is a protein coding gene, while other gene sequences are noncoding sequences. However, in recent years, more studies have shown that some RNA regions (including lncRNA, 5′UTR, 3′UTR, circRNA, etc.) that are traditionally considered not to encode proteins can translate some peptides with a length of less than 100 amino acids. These peptides, of less than 100 amino acids in length, also play a variety of important roles in organisms, including ontogeny, muscle contraction, and DNA repair. Because translatomics is used to sequence and quantify the RNA molecules being translated, it can provide direct translation evidence for these noncoding sequences, and help to find and identify new unknown peptides.

## Conclusion

In conclusion, this study analyzed the process of screening cashmere fineness functional genes by translatomics through Ribo-seq sequencing, found that the *COL6A5* gene may play an important role in cashmere fineness regulation, and provided some theoretical basis for future research on this gene in the field of cashmere fineness regulation.

## Data Availability

The datasets presented in this study can be found in online repositories. The names of the repository/repositories and accession number(s) can be found below: GEO Database, GSE186959.
